# Diagnostic codes of cancer in Skåne healthcare register: a validation study using individual-level data in southern Sweden

**DOI:** 10.1186/s12885-021-08481-5

**Published:** 2021-06-30

**Authors:** Qing Shen, Maria E. C. Schelin, Fang Fang, Anna Jöud

**Affiliations:** 1 Department of Medical Epidemiology and Biostatistics, Karolinska Institutet, Box 210, 171 77 Stockholm, Sweden; 2 Department of Clinical Sciences Lund, Institute for Palliative Care, Lund University, SE-221 00 Lund, Sweden; 3 Unit of Integrative Epidemiology, Institute of Environmental Medicine, Karolinska Institutet, SE-171 77 Stockholm, Sweden; 4 Department of Laboratory medicine, Division of Occupational and Environmental Medicine, Lund University, SE-221 00 Lund, Sweden

**Keywords:** Cancer diagnosis, Register-based, Validity, Healthcare

## Abstract

**Background:**

The Swedish healthcare is decentralised to 21 regions. Detailed information on all delivered care in the southernmost region, Skåne, is prospectively collected in the Skåne Healthcare Register (SHR). The data is updated daily and hence a good source for epidemiological studies. However, the diagnostic codes used to identify cancer patients in SHR have not yet been validated.

**Methods:**

We conducted a validation study including 1,473,204 residents in Skåne region during 2005–2014, with at least one physical consultation in SHR. Newly diagnosed cancer from the Swedish Cancer Register was considered the ‘gold standard’ reference. We estimated the positive predictive value (PPV), sensitivity, and area under the curve (AUC) of a cancer diagnosis based on SHR by level of consultation, for any cancer, and for different cancer types.

**Results:**

There were 61,693 cancers from the Swedish Cancer Register, and 87,650 cancers from SHR. The PPV of SHR-based diagnosis of any cancer was 63.76% (95% confidence interval (CI): 63.44–64.08%) with a sensitivity of 90.58% (95% CI: 90.35–90.81%). The AUC was 0.94, for any cancer. The measures of PPV, sensitivity and AUC varied across levels of care and were higher in specialized care than in primary care. The highest PPV was observed for specialist inpatient care in SHR (89.17, 95% CI 88.89–89.45%) whereas the highest sensitivity was observed for specialized outpatient care in SHR (86.39, 95%CI 86.12–86.66%). Robust validity was noted among most cancers, except for cancers of soft tissues, central nervous system and eye, and endocrine glands.

**Conclusions:**

Our study supports that SHR is a valid and robust healthcare register for cancer diagnosis, with varying validities across levels of care and cancer types. This makes SHR a useful data source for cancer epidemiological studies, especially because the data covers the entire cancer care pathways without time lags for further linkage.

**Supplementary Information:**

The online version contains supplementary material available at 10.1186/s12885-021-08481-5.

## Background

Swedish population and health registers provide a unique opportunity in medical research because of their longitudinal and complete coverage on the national or a regional population [1]. In Sweden, the quality of nationwide registers, including Swedish Cancer Register, Patient Register and Causes of Death Register, are known to be high, with data collected prospectively and independently [2–4]. The utilization of such national healthcare data has however been limited to specialized care, due to the fact that data on primary care has not yet been centralized.

Because the healthcare system in Sweden is organized, governed, and financed at a regional level, there are administrative healthcare databases regionally. One example is the regional database of the southern county of Sweden (the region of Skåne): the Skåne Healthcare Register (SHR). The SHR covers all the healthcare consultations provided in Skåne for the entire population and has been used for descriptive, etiological and health economic research since early 2000 [5]. However, few studies have been performed using the diagnostic codes to ascertain diseases, to follow disease course across levels of care, and to utilize healthcare information at primary care level. Further, studies have been predominantly focused on musculoskeletal disorders and rheumatic diseases, [6–8] leaving the potential to expand the utility of SHR in a broader research area largely unraveled. The diagnoses in the SHR were reported to be valid for psoriatic arthritis, with lower positive predictive value (PPV) in primary care than in secondary care [6]. Other than that, the diagnostic codes in the SHR to ascertain other patients, e.g. patients with cancer, are yet to be validated. Chronic diseases, comorbidities and early identification of suspicious symptoms are increasingly managed in primary care settings. It is therefore important that all healthcare delivery is measured with reliability and consistency [9].

The Swedish Cancer Register collects the nationwide reporting of new cancer cases once a year. While the validity is high, the time-lag limits its usefulness in studies requiring timely update. The SHR is updated daily and is currently used to some extent for follow-up of quality of care. The validity of such data source could therefore promote quality assurance, both in Skåne and other regions. In this study, we aimed to assess the validity of cancer diagnoses in the SHR, using the Swedish Cancer Register as the ‘gold standard’ reference. In addition, we intended to examine the validity of cancer diagnoses by different levels of care (primary care, specialist outpatient and inpatient care) as well as by cancer sites.

## Methods

### Study setting

This study targeted the whole population living in Skåne region, Sweden, during 2005–2014. The Skåne region is the southernmost region of Sweden and has a population around 1.4 million (one-eighth of the Swedish total population) [10]. All Swedish inhabitants have been uniquely assigned an identification number which can be used to link individuals across registers. We identified in total 1,473,204 inhabitants of the Skåne region during the study period, from the Total Population Register, who were also free of cancer diagnosis before 2005, according to the Swedish Cancer Register.

### Data source

SHR has data on all primary care and specialized outpatient and inpatient care continuously updated for all residents living in Skåne region since 1998 onward [5]. Information includes personal identification number, age, sex, type of healthcare, date of consultation, diagnostic codes, as well as codes for surgical and non-surgical treatments. Healthcare in Sweden is predominantly publicly financed and private care is considered to compose a small to negligible proportion of all healthcare in Sweden [5]. In Skåne, reporting to the SHR forms the basis for economic reimbursement to the healthcare providers. Thus, the reporting to SHR from healthcare providers is assumed to be highly motivated if not mandatory. In the present study we defined the first primary diagnosis of any cancer in the SHR to be the diagnostic date, regardless of level of healthcare, during the period 1st January 2005 to 31st December 2014.

The Swedish Cancer Register provides a complete registration of all diagnoses of malignancies in Sweden as early as from 1958, and the completeness of cancer registration has been verified to be 96.3% [2]. A cancer report to the Swedish Cancer Register is required for every cancer diagnosed at clinical, morphological and other laboratory examinations, and those diagnosed at autopsy. The date of diagnosis in Swedish Cancer Register is the date when the cancer diagnosis is established clinically and/or by morphological examination. Usually, patient is informed about the diagnosis after the date of diagnosis reported to the Cancer Register. From the Swedish Cancer Register, we identified all individuals living in Skåne region who received a primary cancer diagnosis of any type during the same study period, excluding cancers detected at autopsy.

Cancer diagnoses were coded with International Classification of Disease 7th revision (ICD-7) in the Swedish Cancer Register and in ICD-10 in the SHR during the study period. The comparison of cancer cases between the SHR and the Swedish Cancer Register was performed by cancer groups. Diagnostic codes for all cancers and cancer classification are listed in Table 1.

**Table 1:** Cancer identification and classification using ICD-7 and ICD-10 codes

Cancer types	ICD-7	ICD-10
Cancers of Lip, oral cavity and pharynx	140–148	C00-C14
Cancers of digestive organs	150–159	C15-C26
Esophagus	150	C15
Colorectal	153–154	C18-C21
Liver	155–156	C22-C24
Pancreas	157	C25
Cancers of Lung and thorax	160–165	C30-C39
Bronchus and lung	162–163	C34
Bone cancers	196	C40-C41
Skin cancers	190–191	C43-C44
Melanoma skin cancer	190	C43
Non-melanoma skin cancer	191	C44
Cancers of soft tissues	197	C45-C49
Breast cancers	170	C50
Other female genital cancers	171–176	C51-C58
Uterus	172	C54
Male genital cancers	177–179	C60-C63
Prostate	177	C61
Cancers of urinary tract	180–181	C64-C68
Kidney	180	C64-C65
Bladder	181	C67-C68
Cancers of central nervous system and eye	192–193	C69-C72
Cancers of endocrine glands	194–195	C73-C75
Hematologic malignancies	200–207	C81-C96
Others	198,199	C76-C80, C97

### Validation of diagnostic codes for cancer

**Fig. 1:**
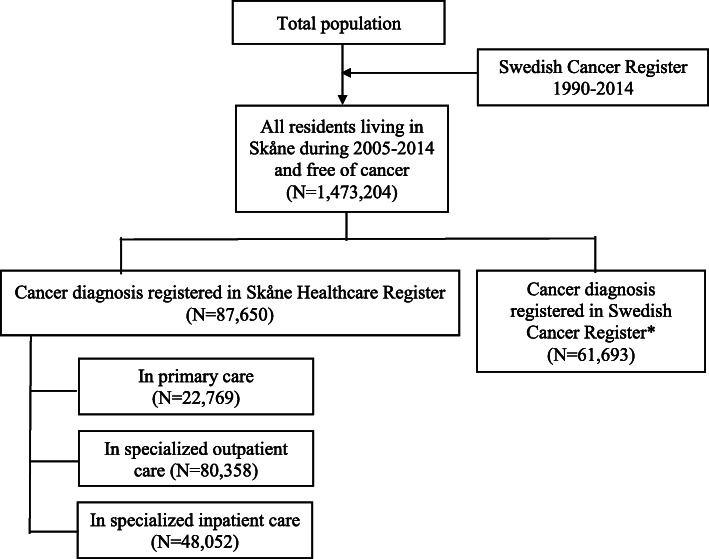
Flowchart of newly diagnosed cancer patients from the Skåne Healthcare Register (SHR) and the Swedish Cancer Register during 2005–2014. *We included only diagnoses of malignancy from the cancer register (27.8% were benign tumor and excluded). We kept only the first malignancy if multiple diagnoses were identified (16%)

Validation of diagnostic codes was performed for patients with a cancer diagnosis in the SHR, using diagnoses from the Swedish Cancer Register as the ‘gold standard’ reference. In the SHR, we defined the first main diagnosis with any cancer to be the SHR cancer diagnosis. Prevalent cancer cases receiving cancer diagnosis before the study period were excluded. From the Swedish Cancer Register, we defined cancer diagnoses as any diagnosis of malignancy, since the reporting of benign tumor was not complete (27.8% were benign tumor and were not included). We used only the first malignancy if multiple diagnoses were identified (16%). In total, we had 87,650 newly diagnosed cancer patients from the SHR and 61,693 newly diagnosed cancer patients from the Swedish Cancer Register. To assess the validity of diagnoses in different levels of care, we further separately ascertained the cancer diagnoses in SHR according to, 1) primary care, 2) specialized outpatient care, and 3) specialized inpatient care (Fig. 1). Patients can be registered multiple times for the same diagnosis at different levels of care.

### Statistical analysis

We calculated the PPV, as the proportion of the patients with cancer diagnosis in the SHR who were confirmed by a diagnosis in the Swedish Cancer Register. Sensitivity was computed as the proportion of cancer patients with cancer diagnosis in SHR, divided by all cancer patients from the Swedish Cancer Register. We calculated false positive rate, denoting the proportion of individuals with cancer diagnosis in the SHR but not in the Swedish Cancer Register, and as well calculated false negative rate, showing the proportion of individuals with cancer diagnosis in the Cancer Register while not in the SHR. Area under receiver operating characteristic curve (AUC) was estimated for assessing the overall robustness of validity in cancer diagnosis in the SHR. The AUC is a scale calculated from sensitivity and specificity with a range from 0.5 to 1.0. An AUC of 0.7–0.8 is considered acceptable, 0.8–0.9 robust and over 0.9 very robust [11]. We firstly calculated PPV, sensitivity, AUC, false positive rate, false negative rate and their 95% confidence intervals (CIs) on cancer patients using all levels of healthcare visits, and then separately by ever having cancer diagnosis according to levels of care (primary care, specialized outpatient care and specialized inpatient care). We also calculated PPV, sensitivity, AUC, false positive rate, false negative rate according to levels of care with priority, with cancer diagnosis to be identified firstly from specialized inpatient care, otherwise from specialized outpatient care or from primary care, exclusively. Cancer-specific calculation was as well provided. To evaluate the changes over time, we further calculated PPV and sensitivity according to calendar year of diagnosis in the SHR. Discrepancy in dates of cancer diagnosis in the SHR compared to Swedish Cancer Register was plotted in histograms, to visualize the difference in time between registered date of diagnosis (in the Cancer Register) and the date when patients were possibly informed of a cancer diagnosis (in the SHR).

All data management and analyses were performed in SAS 9.4 (SAS Institute) and STATA 16.0 (StataCorp LP, College Station, USA). We used Wilson’s score to estimate 95% CIs for all the proportions.

## Results

There were 61,693 newly diagnosed cancers recorded in the Swedish Cancer Register, and 87,650 in the SHR, among which 22,769 patients ever had cancer diagnosed in primary care, 80,358 patients ever had cancer diagnosed in specialized outpatient care and 48,052 ever in specialized inpatient care. The overall PPV of any cancer in the SHR was 63.76% (95% CI 63.44–64.08%), sensitivity was 90.58% (95% CI 90.35–90.81%) (Table 2). The overall false positive rate was 2.25% (2.23–2.28%), and false negative rate was 9.42 (9.19–9.65%) (Supplementary appendix Table S1). AUC for any cancer in all levels of health care was 0.94. The PPV was highest in specialized inpatient care (89.17, 88.89–89.45%) while the highest sensitivity was noted in specialized outpatient care (86.39, 86.12–86.66%). When prioritizing level of care according to specialist inpatient care>specialist outpatient care>primary care, most cancer patients (*N* = 48,052, 54.82% of all cancer patients in the SHR) could be identified in specialized inpatient care, while 40.91% cancer patients were identified in specialized outpatient care and 4.26% were only identified in primary care. The high levels of PPV, sensitivity and AUC were all noted in specialized inpatient care.

**Table 2:** Positive Predictive value (PPV), sensitivity, AUC (area under the curve) and their 95% confidence intervals (CI) of all cancer patients according to level of healthcare, using the Swedish Cancer Register as ‘gold standard’ reference

	Number of cancer patients in SHR	PPV (95%CI), %	Sensitivity (95%CI), %	AUC (95%CI), a scale ranging from 0.5–1.0
All levels of health care^a^	87,650	63.76 (63.44–64.08)	90.58 (90.35–90.81)	0.94 (0.94–0.94)
By level of health care, according to first diagnosis in each level^b^				
Primary care	22,769	70.05 (69.45–70.64)	25.85 (25.51–26.20)	0.63 (0.63–0.63)
Specialized outpatient care	80,358	66.32 (66.00–66.65)	86.39 (86.12–86.66)	0.92 (0.92–0.92)
Specialized inpatient care	48,052	89.17 (88.89–89.45)	69.46 (69.09–69.82)	0.85 (0.84–0.85)
By level of health care, according to priority of care^c^				
1) Specialized inpatient care	48,052	89.17 (88.89–89.45)	69.46 (69.09–69.82)	0.85 (0.84–0.85)
2) Specialized outpatient care	35,861	35.16 (34.67–35.66)	20.44 (20.12–20.76)	0.59 (0.59–0.60)
3) Primary care	3737	11.37 (10.37–12.43)	0.69 (0.63–0.76)	0.50 (0.50–0.50)

^a^If multiple records, we counted the first primary diagnosis of cancer for each patient

^b^Patients could be counted once at each level of care, and three times at maximum among all levels of care

^c^Maximum one time per patient. The priority was given to: inpatient specialist care > outpatient specialist care > primary care

The PPVs and sensitivity also varied across cancer types. The PPV was higher for patients with breast cancer (87.31, 95% CI 86.62–87.98%), male genital cancers (89.43, 95% CI 88.84–90.00%), cancers of urinary tract (83.65, 95% CI 82.57–84.70%) and cancers of lung and thorax (84.64, 95% CI 83.61–85.63%) (Table 3). The lowest PPV was observed for non-melanoma skin cancers (11.50, 95% CI 11.09–11.91%). We found a higher sensitivity among patients with breast cancer (93.74, 95% CI 93.21–94.25%), other female genital cancers (88.61, 95% CI 87.42–89.72%), male genital cancers (88.06, 95% CI 87.45–88.66%) and cancers of urinary tract (87.41, 95% CI 86.40–88.36%). The lowest sensitivity value was noted for patients with cancers of endocrine glands (27.69, 95% CI 25.54–29.92%). The false positive rate was low for all cancer types, with relatively higher false positive rate noted for non-melanoma skin cancer (Supplementary appendix Table S2). The higher false negative rate was observed for cancers of soft tissue, and cancers of central nervous system and eye. The AUC in most cancer types was above threshold of robustness (> 0.8), except for cancers of soft tissues, central nervous system and eye, and endocrine glands. We found an overall higher PPV, sensitivity and AUC in specialized outpatient and inpatient care, than that in primary care (Supplementary appendix Table S3-S5).

**Table 3:** Predictive value (PPV), Sensitivity, AUC (area under the curve) and their 95% confidence intervals (CI) of all cancer patients and by cancer types, using the Swedish Cancer Register as ‘gold standard’ reference

	Cancer Register	Skåne Healthcare Register			
	Number of cancer patients^a^	Number of cancer patients^a^	PPV (95%CI), %	Sensitivity (95%CI), %	AUC (95%CI)
All cancers	61,693	87,650	63.76 (63.44–64.08)	90.58 (90.35–90.81)	0.94 (0.94–0.94)
Cancers of lip, oral cavity and pharynx	1142	1298	72.85 (70.21–75.38)	74.96 (72.34–77.45)	0.87 (0.86–0.89)
Cancers of digestive organs	10,549	11,877	80.13 (79.38–80.86)	85.99 (85.31–86.65)	0.93 (0.93–0.93)
Esophagus	523	465	89.03 (85.83–91.72)	79.16 (75.42–82.56)	0.90 (0.88–0.91)
Colorectal	6837	6774	88.22 (87.43–88.98)	87.41 (86.60–88.18)	0.94 (0.93–0.94)
Liver	902	1059	55.43 (52.38–58.45)	65.08 (61.87–68.19)	0.83 (0.81–0.84)
Pancreas	930	1432	53.49 (50.87–56.10)	82.37 (79.76–84.76)	0.91 (0.90–0.92)
Cancers of lung and thorax	5244	5381	84.64 (83.61–85.63)	80.68 (79.59–81.74)	0.90 (0.90–0.91)
Bronchus and lung	4916	4502	86.34 (85.30–87.33)	79.07 (77.90–80.20)	0.90 (0.89–0.90)
Bone cancer	95	240	43.40 (35.57–51.48)	72.63 (62.52–81.28)	0.86 (0.82–0.91)
Skin cancer	7434	29,371	20.08 (19.60–20.56)	73.94 (72.93–74.94)	0.86 (0.86–0.87)
Melanoma skin cancer	3104	3267	74.64 (73.07–76.16)	74.81 (73.24–76.33)	0.87 (0.87–0.88)
Non-melanoma skin cancer	4330	26,104	11.50 (11.09–11.91)	63.28 (61.82–64.72)	0.81 (0.80–0.82)
Cancers of soft tissues	361	797	52.74 (47.73–57.71)	58.73 (53.45–63.85)	0.79 (0.77–0.82)
Breast cancer	8681	9451	87.31 (86.62–87.98)	93.74 (93.21–94.25)	0.97 (0.97–0.97)
Other female genital cancers	3029	3476	81.70 (80.34–83.01)	88.61 (87.42–89.72)	0.94 (0.94–0.95)
Uterus	1293	1166	92.28 (90.60–93.75)	83.22 (81.07–85.22)	0.92 (0.91–0.93)
Male genital cancers	11,318	11,356	89.43 (88.84–90.00)	88.06 (87.45–88.66)	0.94 (0.94–0.94)
Prostate	10,748	10,421	90.53 (89.95–91.08)	87.77 (87.14–88.39)	0.94 (0.94–0.94)
Cancers of urinary tract	4526	4937	83.65 (82.57–84.70)	87.41 (86.40–88.36)	0.94 (0.93–0.94)
Kidney	1224	1402	74.04 (71.66–76.32)	84.80 (82.67–86.77)	0.92 (0.91–0.93)
Bladder	3302	3219	87.57 (86.38–88.69)	85.37 (84.12–86.56)	0.93 (0.92–0.93)
Cancers of central nervous system and eye	1712	1452	66.81 (64.24–69.31)	53.15 (50.76–55.54)	0.77 (0.75–0.78)
Cancers of endocrine glands	1647	671	73.19 (69.53–76.64)	27.69 (25.54–29.92)	0.64 (0.63–0.65)
Hematologic malignancies	4117	4779	75.45 (74.17–76.69)	84.11 (82.96–85.22)	0.92 (0.91–0.93)
Others	1838	2564	41.69 (39.04–44.38)	30.58 (28.48–32.74)	0.65 (0.64–0.66)

^a^ If multiple records, we counted the first primary diagnosis of cancer for each patient

**Fig. 2:**
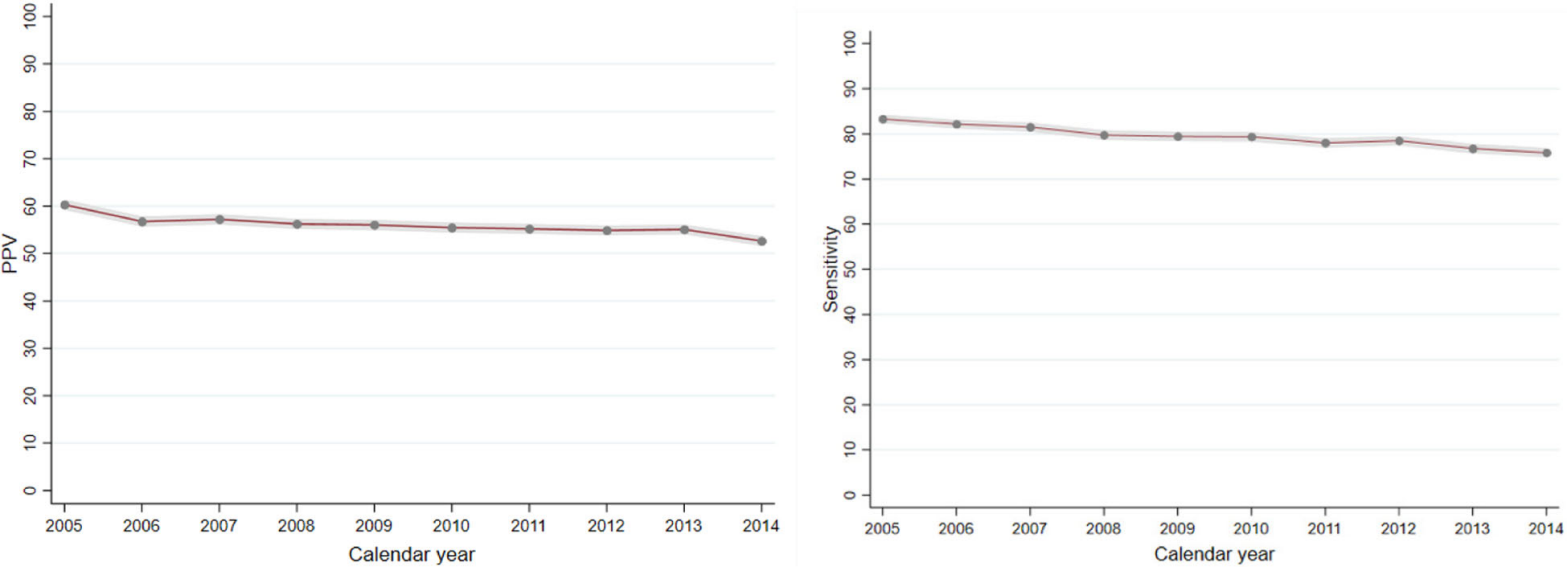
Positive predictive value (PPV) and sensitivity of all cancer diagnosis according to calendar year, using the Swedish Cancer Register as ‘gold standard’ reference

During the study period from 2005 to 2014, there was a slightly decline over time in the PPVs (*P* for trend < 0.001) and sensitivity (*P* for trend < 0.001) (Fig. 2).

**Fig. 3:**
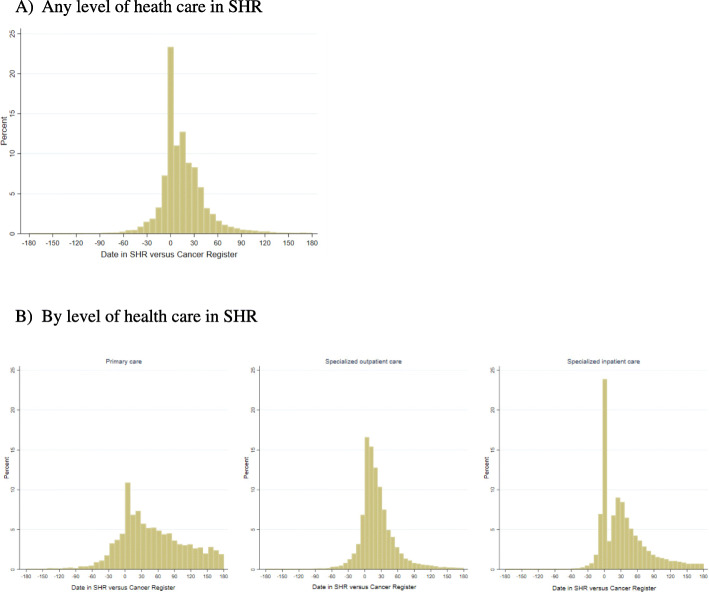
Difference in dates between the date of cancer diagnosis in SHR and the date of diagnosis in Cancer Register, using date of diagnosis in Cancer Register as date “0”

When evaluating the difference in diagnostic dates between the SHR and the Swedish Cancer Register, patients might receive a cancer diagnosis in the SHR both before and after the diagnostic dates registered in the Cancer Register (Fig. 3). More patients received a cancer diagnosis in the SHR later than the date registered in the Cancer Register, and more than 20% patients had the same dates in both registers. When separately accessing the difference by level of care, we found that patients who received a cancer diagnosis from the specialized inpatient care were more likely to have the same diagnostic date, as registered in the Swedish Cancer Register. Cancer diagnosis in primary care were more likely to be recorded after the date of diagnosis from the Swedish Cancer Register.

## Discussion

In this validation study, we compared all cancer diagnoses recorded in the population-based SHR with the diagnoses in the Swedish Cancer Register, and found a moderate-to-high validity of cancer diagnoses in the SHR. The validity measures varied across levels of care and were higher in specialized care than in primary care. We found in general a robust validity among most cancers, except for cancers of soft tissues, central nervous system and eye, and endocrine glands. A large variation in the PPV and sensitivity for different cancer sites was noted, with higher levels of PPV and sensitivity observed for patients with breast cancer, female and male genital cancers, as well as urinary tract cancers. Differences in dates of diagnoses in the two data sources were found to vary depending on level of care. These findings indicate the SHR to be a robust and valid healthcare register for studying cancer in general and several cancer types specifically. It can also be used in addition to the Cancer Register, given the timely update of data as well as the availability of health care utilization data across all levels of care.

The SHR represents a unique and valuable source of healthcare data [5]. It entails information on all levels of care as well as all types of healthcare professionals, and facilitates studies comparing healthcare services provided by physicians, nurses, physiotherapists, social workers, etc. Studies investigating the flow of care and diagnostic process of specific patient groups, including cancer patients, are of crucial importance for disease differentiation, treatment, follow up and supportive care after treatment, providing basis for integrating standardized care processes [12]. With the improvement in cancer survival, growing numbers of people will be living with cancer as a chronic disease, [13] emphasizing the need to provide systematic monitoring to cancer patients and survivors. It is of importance that the SHR integrates cross-level healthcare data including primary care, because most chronic conditions are handled in primary care settings [9, 14].

In our study, we found overall 64% of the cancer patients diagnosed in the SHR to be confirmed with a cancer using the Cancer Register as standard of reference, while 91% cancer patients recorded in the Cancer Register were recorded also in the SHR. The AUC is 0.94 for any cancer in all levels of health care, suggesting the SHR to be a robust database for identifying the majority of cancer patients. Meanwhile, 2.25% ‘misdiagnosed’ individuals in the SHR were noted - patients who never received a cancer diagnosis in the Cancer Register. It is not uncommon to observe non-cancer individuals to be diagnosed with a cancer in initial diagnostic evaluation. Pre-diagnostic evaluation was mostly initiated in primary care settings [14]. For individuals with suspected cancer symptoms, there was investigation and referral decisions, to differentiate cancer from other, benign conditions. Further, we found a variety of PPVs of cancer diagnoses in SHR across levels of care, with highest PPV noted in specialized inpatient care. This finding was expected since patients were often diagnosed and treated in specialized care, while primary care might have been responsible for the first referral of uncertain symptoms and then, later, for the follow-up. Higher levels of PPV and sensitivity were observed for several cancers, including breast cancer, female and male genital cancers, and urinary tract cancers, than for other cancers. These findings might be due to the easily accessible screening techniques for breast cancer, cervical cancer and prostate cancer [15–17]. Lower level of PPV and sensitivity, and higher level of false positive were expected for non-melanoma skin cancer, due to the fact that basal cell carcinoma, one common type of non-melanoma skin cancer, was not reported to Swedish Cancer Register [2].

During the study period, there was a slightly decline trend over time for PPV and sensitivity. One possibility for the declining trend is due to the inclusion of health professionals other than physicians to be allowed to register a diagnosis in the SHR [5]. We found that date of cancer diagnosis in primary care was more likely to be registered after the date from Cancer Register. This was in line with our hypothesis, as patients suspected for a potential cancer were more likely to be diagnosed and treated in specialized care and later referred back to primary care for long-term monitoring. The ability of primary care to provide coordinated and comprehensive care for cancer survivors is important, since the responsibility for post-treatment care has largely been distributed to primary care physicians [18]. In addition to short-term effects from cancer treatment, cancer survivors remain at risk of relapse, development of secondary cancer, and long-term morbidity related to the disease and its treatment [19]. This highlights a potential of using the SHR in investigating cancer pre-diagnostic symptoms, disease course, long-term health outcomes as well as healthcare service utilization.

The use of regional healthcare databases has been suggested as complement sources of data to national registries. The latest validation of the Swedish Cancer Register was conducted using a sample survey of medical records from more than 20 years ago [2]. There was an overall underreporting of estimated 3.7% of cancer patients, [2] indicating a decline in completeness comparing to the prior review of less than 2% [20]. Therefore it is hard to draw inference about the completeness of the Cancer Register in recent years. Validation work should ideally be performed at relatively short and regular intervals for better quality assessment in healthcare organizations, and also for the large number of studies that are dependent on the reporting of such a register. Further, even though the completeness of the cancer diagnoses in the Cancer Register was overall very high, some variations were noted on specific sites. For instance, the underreporting was about 15% for hematological malignancies [21] and more than 20% for pancreatic cancer [22]. The registration of non-melanoma skin cancer was also incomplete [23]. It is therefore a potential to use the SHR as a supplement source of cancer cases to the Cancer Register, when the impact of underreporting for certain cancers is known to be substantial. Moreover, the timely update of the SHR on regular basis, in contrast to the Cancer Register which is updated once a year and often requires further linkage, facilitates access to the most recent data, depending on research context and question of interest.

The major strength of this study includes the use of Swedish Cancer Register as ‘gold standard’ reference for validation, which has almost complete and accurate records on cancer diagnoses across the country. The SHR has prospectively and independently collected information on all healthcare visits and has an almost complete coverage of health consultations in different levels of health care. Because healthcare is largely financed by the universal healthcare insurance to each resident in Sweden, the findings of the present study are not likely greatly influenced by other factors such as socioeconomic status.

Our study has some limitations. Firstly, we ascertained the first primary cancer diagnosis in SHR as the date of cancer diagnosis. It was possible that a cancer diagnosis was recorded in SHR when individuals were having suspected cancer symptoms or receiving cancer treatment, leaving the date of diagnosis arbitrarily defined. This is less likely to have impact when the research aim is to study long-term health outcomes of cancer survivors, where the exact date of cancer diagnosis is less influential. Secondly, the SHR does not contain healthcare provided in nursing homes. This would lead to a small missing number of cancer cases in SHR among the elderly population, since people living in nursing homes are in advanced age, often with comorbidities and at risk of being diagnosed with cancer that are not further treated at specialized care. Cancers diagnosed in nursing homes that warrant further treatment at hospital would however naturally be recorded in SHR at the hospital. Further, information on tumour stage and clinical characteristics of the cancer patients was largely unavailable in the SHR and therefore not studied in detail.

## Conclusion

Our study supports that the SHR is a valid and robust healthcare register for several cancer diagnoses, with variations noted across level of care. This makes SHR a useful data source for epidemiological studies for cancer, particularly for some cancer types, because the data does not suffer from time lags and provides data on the entire care pathway and care utilization without further linkage, as opposed to the national cancer register data.

## Supplementary Information

**Additional file 1 Table S1**. False positive rate and false negative rate and their 95% confidence intervals (CI) of all cancer patients according to level of healthcare, using the Swedish Cancer Register as ‘gold standard’ reference. **Table S2**. False positive rate and false negative rate, and their 95% confidence intervals (CI) of all cancer patients and by cancer types, using the Swedish Cancer Register as ‘gold standard’ reference. **Table S3**. Predictive value (PPV), Sensitivity, false positive rate, false negative rate, AUC and their 95% confidence intervals (CI) of all cancer patients and by cancer types in primary care in Skåne region, using the Swedish Cancer Register as ‘gold standard’ reference. **Table S4**. Predictive value (PPV), Sensitivity, false positive rate, false negative rate, AUC and their 95% confidence intervals (CI) of all cancer patients and by cancer types in specialised outpatient care in Skåne region, using the Swedish Cancer Register as ‘gold standard’ reference. **Table S5**. Predictive value (PPV), Sensitivity, false positive rate, false negative rate, AUC and their 95% confidence intervals (CI) of all cancer patients and by cancer types in specialised inpatient care in Skåne region, using the Swedish Cancer Register as ‘gold standard’ reference.

## Data Availability

Data can be accessed upon reasonable request to Anna Jöud at anna.joud@med.lu.se

## Abbreviations

SHR: Skåne Healthcare Register
SCR: Swedish Cancer Register
NPV: Negative predictive value
PPV: Positive predictive value
AUC: Area under the curve
ICD: International Classification of Disease
