# Supplementary material for: Diagnostic codes of cancer in Skåne healthcare register: a validation study using individual-level data in southern Sweden
Source: BMC Cancer. 2021 Jun 30;21:759. doi: 10.1186/s12885-021-08481-5 (PMC8244146; doi:10.1186/s12885-021-08481-5)
Supplement: Supplementary file 1 — Additional file 1 Table S1. False positive rate and false negative rate and their 95% confidence intervals (CI) of all cancer patients according to level of healthcare, using the Swedish Cancer Register as ‘gold standard’ reference. Table S2. False positive rate and false negative rate, and their 95% confidence intervals (CI) of all cancer patients and by cancer types, using the Swedish Cancer Register as ‘gold standard’ reference. Table S3. Predictive value (PPV), Sensitivity, false positive rate, false negative rate, AUC and their 95% confidence intervals (CI) of all cancer patients and by cancer types in primary care in Skåne region, using the Swedish Cancer Register as ‘gold standard’ reference. Table S4. Predictive value (PPV), Sensitivity, false positive rate, false negative rate, AUC and their 95% confidence intervals (CI) of all cancer patients and by cancer types in specialised outpatient care in Skåne region, using the Swedish Cancer Register as ‘gold standard’ reference. Table S5. Predictive value (PPV), Sensitivity, false positive rate, false negative rate, AUC and their 95% confidence intervals (CI) of all cancer patients and by cancer types in specialised inpatient care in Skåne region, using the Swedish Cancer Register as ‘gold standard’ reference. [file 12885_2021_8481_MOESM1_ESM.docx]

**Diagnostic codes of cancer in Skåne Healthcare Register:**

**a validation study using individual-level data in southern Sweden**

Supplementary appendix

Table S1. False positive rate and false negative rate and their 95% confidence intervals (CI) of all cancer patients according to level of healthcare, using the Swedish Cancer Register as ‘gold standard’ reference.

|  | Number of cancer patients in SHR | False positive  (95%CI), % | False negative  (95%CI), % |
| --- | --- | --- | --- |

| All levels of health care^†^ | 87,650 | 2.25 (2.23-2.28) | 9.42 (9.19-9.65) |
| --- | --- | --- | --- |
|  |  |  |  |
| By level of health care, according to first diagnosis in each level^‡^ |  |  |  |
| Primary care | 22,769 | 0.48 (0.47-0.49) | 74.15 (73.80-74.49) |
| Specialized outpatient care | 80,358 | 1.92 (1.89-1.94) | 13.61 (13.34-13.88) |
| Specialized inpatient care | 48,052 | 0.37 (0.36-0.38) | 30.54 (30.18-30.91) |
|  |  |  |  |
| By level of health care, according to priority of care^*^ |  |  |  |
| 1) Specialized inpatient care | 48,052 | 0.37 (0.36-0.38) | 30.54 (30.18-30.91) |
| 2) Specialized outpatient care | 35,861 | 1.65 (1.63-1.67) | 79.56 (79.24-79.88) |
| 3) Primary care | 3,737 | 0.23 (0.23-0.24) | 99.31 (99.24-99.37) |

^†^ If multiple records, we counted the first primary diagnosis of cancer for each patient.

^‡^ Patients could be counted once at each level of care, and three times at maximum among all levels of care.

^*^ Maximum one time per patient. The priority was given to: inpatient specialist care > outpatient specialist care > primary care.

Table S2. False positive rate and false negative rate, and their 95% confidence intervals (CI) of all cancer patients and by cancer types, using the Swedish Cancer Register as ‘gold standard’ reference.

|  | Cancer Register | Skåne Healthcare Register | | |
| --- | --- | --- | --- | --- |
|  | Number of cancer patients^†^ | Number of cancer patients^†^ | False positive  (95%CI), % | False negative  (95%CI) |
| All cancers | 61,693 | 87,650 | 2.25 (2.23-2.28) | 9.42 (9.19-9.65) |
|  |  |  |  |  |
| Cancers of lip, oral cavity and pharynx | 1142 | 1298 | 0.02 (0.02-0.03) | 25.04 (22.55-27.66) |
| Cancers of digestive organs | 10549 | 11877 | 0.16 (0.15-0.17) | 14.01 (13.35-14.69) |
| Esophagus | 523 | 465 | 0.00 (0.00-0.00) | 20.84 (17.44-24.58) |
| Colorectal | 6837 | 6774 | 0.06 (0.05-0.06) | 12.59 (11.82-13.40) |
| Liver | 902 | 1059 | 0.03 (0.03-0.04) | 34.92 (31.81-38.13) |
| Pancreas | 930 | 1432 | 0.05 (0.04-0.05) | 17.63 (15.24-20.24) |
| Cancers of lung and thorax | 5244 | 5381 | 0.05 (0.05-0.06) | 19.32 (18.26-20.41) |
| Bronchus and lung | 4916 | 4502 | 0.04 (0.04-0.05) | 20.93 (19.80-22.10) |
| Bone cancer | 95 | 240 | 0.01 (0.01-0.01) | 27.37 (18.72-37.48) |
| Skin cancer | 7434 | 29371 | 1.55 (1.53-1.57) | 26.06 (25.06-27.07) |
| Melanoma skin cancer | 3104 | 3267 | 0.06 (0.05-0.06) | 25.19 (23.67-26.76) |
| Non-melanoma skin cancer | 4330 | 26104 | 1.49 (1.47-1.51) | 36.72 (35.28-38.18) |
| Cancers of soft tissues | 361 | 797 | 0.01 (0.01-0.02) | 41.27 (36.15-46.55) |
| Breast cancer | 8681 | 9451 | 0.08 (0.08-0.09) | 6.26 (5.75-6.79) |
| Other female genital cancers | 3029 | 3476 | 0.04 (0.04-0.05) | 11.39 (10.28-12.58) |
| Uterus | 1293 | 1166 | 0.01 (0.01-0.01) | 16.78 (14.78-18.93) |
| Male genital cancers | 11318 | 11356 | 0.08 (0.08-0.09) | 11.94 (11.34-12.55) |
| Prostate | 10748 | 10421 | 0.07 (0.07-0.07) | 12.23 (11.61-12.86) |
| Cancers of urinary tract | 4526 | 4937 | 0.05 (0.05-0.06) | 12.59 (11.64-13.60) |
| Kidney | 1224 | 1402 | 0.03 (0.02-0.03) | 15.20 (13.23-17.33) |
| Bladder | 3302 | 3219 | 0.03 (0.03-0.03) | 14.63 (13.44-15.88) |
| Cancers of central nervous system and eye | 1712 | 1452 | 0.03 (0.03-0.04) | 46.85 (44.46-49.24) |
| Cancers of endocrine glands | 1647 | 671 | 0.01 (0.01-0.01) | 72.31 (70.08-74.46) |
| Hematologic malignancies | 4117 | 4779 | 0.08 (0.08-0.08) | 15.89 (14.78-17.04) |
| Others | 1838 | 2564 | 0.06 (0.05-0.06) | 69.42 (67.26-71.52) |

^†^ If multiple records, we counted the first primary diagnosis of cancer for each patient.

Table S3. Predictive value (PPV), Sensitivity, false positive rate, false negative rate, AUC and their 95% confidence intervals (CI) of all cancer patients and by cancer types in primary care in Skåne region, using the Swedish Cancer Register as ‘gold standard’ reference.

|  | Cancer Register | Skåne Healthcare Register | | | | | |
| --- | --- | --- | --- | --- | --- | --- | --- |
|  | Number of cancer patients^†^ | Number of cancer patients^†^ | PPV (95%CI), % | Sensitivity (95%CI), % | False positive  (95%CI), % | False negative  (95%CI) | AUC  (95%CI) |
| All cancers | 61693 | 22769 | 70.05 (69.45-70.64) | 25.85 (25.51-26.20) | 0.48 (0.47-0.49) | 74.15 (73.80-74.49) | 0.63 (0.63-0.63) |
|  |  |  |  |  |  |  |  |
| Cancers of lip, oral cavity and pharynx | 388 | 201 | 83.58 (77.72-88.42) | 43.30 (38.31-48.39) | 0.00 (0.00-0.00) | 56.70 (51.61-61.69) | 0.72 (0.69-0.74) |
| Cancers of digestive organs | 3941 | 3473 | 80.42 (79.06-81.73) | 70.87 (69.42-72.29) | 0.05 (0.04-0.05) | 29.13 (27.71-30.58) | 0.85 (0.85-0.86) |
| Esophagus | 202 | 138 | 91.30 (85.30-95.43) | 62.38 (55.31-69.08) | 0.00 (0.00-0.00) | 37.62 (30.92-44.69) | 0.81 (0.78-0.85) |
| Colorectal | 2534 | 2145 | 88.53 (87.11-89.85) | 74.94 (73.21-76.62) | 0.02 (0.02-0.02) | 25.06 (23.38-26.79) | 0.87 (0.87-0.88) |
| Liver | 371 | 250 | 54.40 (48.01-60.69) | 36.66 (31.74-41.79) | 0.01 (0.01-0.01) | 63.34 (58.21-68.26) | 0.68 (0.66-0.71) |
| Pancreas | 323 | 376 | 51.60 (46.42-56.75) | 60.06 (54.49-65.44) | 0.01 (0.01-0.01) | 39.94 (34.56-45.51) | 0.80 (0.77-0.83) |
| Cancers of lung and thorax | 1780 | 1189 | 82.42 (80.14-84.55) | 55.06 (52.71-57.39) | 0.01 (0.01-0.02) | 44.94 (42.61-47.29) | 0.78 (0.76-0.79) |
| Bronchus and lung | 1727 | 1081 | 84.18 (81.87-86.31) | 52.69 (50.31-55.07) | 0.01 (0.01-0.01) | 47.31 (44.93-49.69) | 0.76 (0.75-0.78) |
| Bone cancer | 40 | 34 | 50.00 (32.43-67.57) | 42.50 (27.04-59.11) | 0.00 (0.00-0.00) | 57.50 (40.89-72.96) | 0.71 (0.63-0.79) |
| Skin cancer | 3054 | 5077 | 24.09 (22.92-25.29) | 40.05 (38.30-41.81) | 0.28 (0.27-0.29) | 59.95 (58.19-61.70) | 0.70 (0.69-0.71) |
| Melanoma skin cancer | 1207 | 729 | 76.13 (72.87-79.18) | 45.98 (43.14-48.84) | 0.01 (0.01-0.01) | 54.02 (51.16-56.86) | 0.73 (0.72-0.74) |
| Non-melanoma skin cancer | 2056 | 4235 | 13.11 (12.10-14.16) | 26.99 (25.08-28.97) | 0.26 (0.26-0.27) | 73.01 (71.03-74.92) | 0.63 (0.62-0.64) |
| Cancers of soft tissues | 169 | 76 | 65.79 (54.01-76.29) | 29.59 (22.82-37.08) | 0.00 (0.00-0.00) | 70.41 (62.92-77.18) | 0.65 (0.61-0.68) |
| Breast cancer | 3179 | 3267 | 85.58 (84.33-86.77) | 87.95 (86.77-89.06) | 0.03 (0.03-0.04) | 12.05 (10.94-13.23) | 0.94 (0.93-0.95) |
| Other female genital cancers | 830 | 679 | 85.13 (82.22-87.72) | 69.64 (66.38-72.75) | 0.01 (0.01-0.01) | 30.36 (27.25-33.62) | 0.85 (0.83-0.86) |
| Uterus | 352 | 200 | 97.00 (93.58-98.89) | 55.11 (49.75-60.39) | 0.00 (0.00-0.00) | 44.89 (39.61-50.25) | 0.78 (0.75-0.80) |
| Male genital cancers | 4141 | 3524 | 85.73 (84.53-86.87) | 72.95 (71.57-74.30) | 0.04 (0.03-0.04) | 27.05 (25.70-28.43) | 0.86 (0.86-0.87) |
| Prostate | 4008 | 3343 | 87.26 (86.08-88.37) | 72.78 (71.37-74.15) | 0.03 (0.03-0.03) | 27.22 (25.85-28.63) | 0.86 (0.86-0.87) |
| Cancers of urinary tract | 1434 | 1148 | 85.89 (83.74-87.85) | 68.76 (66.29-71.15) | 0.01 (0.01-0.01) | 31.24 (28.85-33.71) | 0.84 (0.83-0.86) |
| Kidney | 451 | 416 | 75.00 (70.55-79.09) | 69.18 (64.69-73.41) | 0.01 (0.01-0.01) | 30.82 (26.59-35.31) | 0.85 (0.82-0.87) |
| Bladder | 1036 | 709 | 91.82 (89.55-93.73) | 62.84 (59.81-65.79) | 0.00 (0.01-0.00) | 37.16 (34.21-40.19) | 0.81 (0.80-0.83) |
| Cancers of central nervous system and eye | 1020 | 415 | 63.61 (58.78-68.25) | 25.88 (23.22-28.69) | 0.01 (0.01-0.01) | 74.12 (71.31-76.78) | 0.63 (0.62-0.64) |
| Cancers of endocrine glands | 1234 | 172 | 70.93 (63.53-77.59) | 9.89 (8.28-11.69) | 0.00 (0.00-0.00) | 90.11 (88.31-91.72) | 0.55 (0.54-0.56) |
| Hematologic malignancies | 1419 | 1292 | 72.99 (70.48-75.39) | 66.46 (63.93-68.91) | 0.02 (0.02-0.03) | 33.54 (31.09-36.07) | 0.83 (0.82-0.84) |
| Others | 1157 | 330 | 35.15 (30.00-40.57) | 10.03 (8.36-11.90) | 0.02 (0.02-0.01) | 89.97 (88.10-91.64) | 0.55 (0.54-0.56) |

^†^ If multiple records, we counted the first primary diagnosis of cancer for each patient.

Table S4. Predictive value (PPV), Sensitivity, false positive rate, false negative rate, AUC and their 95% confidence intervals (CI) of all cancer patients and by cancer types in specialised outpatient care in Skåne region, using the Swedish Cancer Register as ‘gold standard’ reference.

|  | Cancer Register | Skåne Healthcare Register | | | | | |
| --- | --- | --- | --- | --- | --- | --- | --- |
|  | Number of cancer patients^†^ | Number of cancer patients^†^ | PPV (95%CI), % | Sensitivity (95%CI), % | False positive  (95%CI), % | False negative  (95%CI) | AUC  (95%CI) |
| All cancers | 61693 | 80358 | 66.32 (66.00-66.65) | 86.39 (86.12-86.66) | 1.92 (1.89-1.94) | 13.61 (13.34-13.88) | 0.92 (0.92-0.92) |
|  |  |  |  |  |  |  |  |
| Cancers of lip, oral cavity and pharynx | 889 | 1135 | 74.71 (72.08-77.22) | 95.39 (93.80-96.67) | 0.02 (0.02-0.02) | 4.61 (3.33-6.20) | 0.98 (0.97-0.98) |
| Cancers of digestive organs | 8941 | 9801 | 85.37 (84.65-86.06) | 93.58 (93.05-94.08) | 0.10 (0.10-0.11) | 6.42 (5.92-6.95) | 0.97 (0.96-0.97) |
| Esophagus | 410 | 430 | 91.40 (88.33-93.87) | 95.85 (93.44-97.57) | 0.00 (0.00-0.00) | 4.15 (2.43-6.56) | 0.98 (0.97-0.99) |
| Colorectal | 5895 | 6079 | 91.25 (90.51-91.95) | 94.10 (93.46-94.68) | 0.04 (0.04-0.04) | 5.90 (5.32-6.54) | 0.97 (0.97-0.97) |
| Liver | 609 | 798 | 63.78 (60.34-67.13) | 83.58 (80.39-86.44) | 0.02 (0.02-0.02) | 16.42 (13.56-19.61) | 0.92 (0.90-0.93) |
| Pancreas | 749 | 1152 | 61.63 (58.75-64.45) | 94.79 (92.95-96.27) | 0.03 (0.03-0.04) | 5.21 (3.73-7.05) | 0.97 (0.97-0.98) |
| Cancers of lung and thorax | 4127 | 4308 | 89.02 (88.05-89.94) | 92.92 (92.10-93.69) | 0.03 (0.03-0.04) | 7.08 (6.31-7.90) | 0.96 (0.96-0.97) |
| Bronchus and lung | 3798 | 3875 | 90.58 (89.62-91.48) | 92.42 (91.53-93.24) | 0.03 (0.02-0.03) | 7.58 (6.76-8.47) | 0.96 (0.96-0.97) |
| Bone cancer | 82 | 135 | 51.11 (42.37-59.81) | 84.15 (74.42-91.28) | 0.00 (0.00-0.01) | 15.85 (8.72-25.58) | 0.92 (0.88-0.96) |
| Skin cancer | 6930 | 25275 | 21.06 (20.56-21.57) | 76.81 (75.80-77.80) | 1.42 (1.40-1.44) | 23.19 (22.20-24.20) | 0.88 (0.87-0.88) |
| Melanoma skin cancer | 2591 | 2959 | 77.02 (75.46-78.52) | 87.96 (86.64-89.19) | 0.05 (0.05-0.05) | 12.04 (10.81-13.36) | 0.94 (0.93-0.95) |
| Non-melanoma skin cancer | 3914 | 21886 | 11.94 (11.52-12.38) | 66.79 (65.29-68.26) | 1.37 (1.36-1.39) | 33.21 (31.74-34.71) | 0.83 (0.82-0.83) |
| Cancers of soft tissues | 246 | 369 | 55.83 (50.59-60.96) | 83.74 (78.52-88.12) | 0.01 (0.01-0.01) | 16.26 (11.88-21.48) | 0.92 (0.90-0.94) |
| Breast cancer | 8228 | 8869 | 90.72 (90.10-91.32) | 97.79 (97.45-98.09) | 0.06 (0.06-0.06) | 2.21 (1.91-2.55) | 0.99 (0.99-0.99) |
| Other female genital cancers | 2648 | 3022 | 84.02 (82.66-85.31) | 95.88 (95.06-96.61) | 0.03 (0.03-0.04) | 4.12 (3.39-4.94) | 0.98 (0.98-0.98) |
| Uterus | 1052 | 1088 | 92.74 (91.03-94.21) | 95.91 (94.53-97.03) | 0.01 (0.00-0.01) | 4.09 (2.97-5.47) | 0.98 (0.97-0.99) |
| Male genital cancers | 10602 | 10783 | 91.35 (90.80-91.87) | 92.91 (92.40-93.39) | 0.07 (0.06-0.07) | 7.09 (6.61-7.60) | 0.96 (0.96-0.97) |
| Prostate | 10065 | 10131 | 92.01 (91.47-92.54) | 92.62 (92.09-93.12) | 0.06 (0.05-0.06) | 7.38 (6.88-7.91) | 0.96 (0.96-0.97) |
| Cancers of urinary tract | 4092 | 4487 | 85.56 (84.50-86.57) | 93.82 (93.04-94.54) | 0.05 (0.04-0.05) | 6.18 (5.46-6.96) | 0.97 (0.97-0.97) |
| Kidney | 1059 | 1279 | 77.72 (75.33-79.97) | 93.86 (92.24-95.23) | 0.02 (0.02-0.02) | 6.14 (4.77-7.76) | 0.97 (0.96-0.98) |
| Bladder | 2944 | 3105 | 88.60 (87.43-89.70) | 93.44 (92.49-94.31) | 0.03 (0.02-0.03) | 6.56 (5.69-7.51) | 0.97 (0.96-0.97) |
| Cancers of central nervous system and eye | 1463 | 1097 | 74.02 (71.32-76.59) | 55.50 (52.91-58.07) | 0.02 (0.02-0.02) | 44.50 (41.93-47.09) | 0.78 (0.76-0.79) |
| Cancers of endocrine glands | 1390 | 544 | 76.10 (72.29-79.63) | 29.78 (27.39-32.27) | 0.01 (0.01-0.01) | 70.22 (67.73-72.61) | 0.65 (0.64-0.66) |
| Hematologic malignancies | 3496 | 4173 | 78.46 (77.18-79.70) | 93.65 (92.79-94.44) | 0.06 (0.06-0.07) | 6.35 (5.56-7.21) | 0.97 (0.96-0.97) |
| Others | 960 | 948 | 48.84 (45.61-52.07) | 48.23 (45.03-51.44) | 0.04 (0.03-0.04) | 51.77 (48.56-54.97) | 0.74 (0.73-0.76) |

^†^ If multiple records, we counted the first primary diagnosis of cancer for each patient.

Table S5. Predictive value (PPV), Sensitivity, false positive rate, false negative rate, AUC and their 95% confidence intervals (CI) of all cancer patients and by cancer types in specialised inpatient care in Skåne region, using the Swedish Cancer Register as ‘gold standard’ reference.

|  | Cancer Register | Skåne Healthcare Register | | | | | |
| --- | --- | --- | --- | --- | --- | --- | --- |
|  | Number of cancer patients^†^ | Number of cancer patients^†^ | PPV (95%CI), % | Sensitivity (95%CI), % | False positive  (95%CI), % | False negative  (95%CI) | AUC  (95%CI) |
| All cancers | 61693 | 48052 | 89.17 (88.89-89.45) | 69.46 (69.09-69.82) | 0.37 (0.36-0.38) | 30.54 (30.18-30.91) | 0.85 (0.84-0.85) |
|  |  |  |  |  |  |  |  |
| Cancers of lip, oral cavity and pharynx | 839 | 775 | 93.29 (91.29-94.95) | 86.17 (83.65-88.44) | 0.00 (0.00-0.00) | 13.83 (11.56-16.35) | 0.93 (0.92-0.94) |
| Cancers of digestive organs | 9098 | 10130 | 83.65 (82.92-84.37) | 93.14 (92.60-93.65) | 0.12 (0.11-0.12) | 6.86 (6.35-7.40) | 0.97 (0.96-0.97) |
| Esophagus | 399 | 416 | 90.38 (87.14-93.04) | 94.24 (91.48-96.31) | 0.00 (0.00-0.00) | 5.76 (3.69-8.52) | 0.97 (0.96-0.98) |
| Colorectal | 6001 | 6118 | 91.73 (91.01-92.41) | 93.52 (92.87-94.13) | 0.04 (0.03-0.04) | 6.48 (5.87-7.13) | 0.97 (0.96-0.97) |
| Liver | 635 | 921 | 58.52 (55.26-61.73) | 84.88 (81.86-87.58) | 0.03 (0.02-0.03) | 15.12 (12.42-18.14) | 0.92 (0.91-0.94) |
| Pancreas | 754 | 1305 | 55.02 (52.27-57.74) | 95.23 (93.45-96.63) | 0.04 (0.04-0.05) | 4.77 (3.37-6.55) | 0.98 (0.97-0.98) |
| Cancers of lung and thorax | 3999 | 4152 | 88.46 (87.45-89.42) | 91.85 (90.96-92.68) | 0.03 (0.03-0.04) | 8.15 (7.32-9.04) | 0.96 (0.95-0.96) |
| Bronchus and lung | 3698 | 3805 | 88.99 (87.95-89.97) | 91.56 (90.62-92.44) | 0.03 (0.03-0.03) | 8.44 (7.56-9.38) | 0.96 (0.95-0.96) |
| Bone cancer | 75 | 93 | 67.74 (57.25-77.07) | 84.00 (73.72-91.45) | 0.00 (0.00-0.00) | 16.00 (8.55-26.28) | 0.92 (0.88-0.96) |
| Skin cancer | 3643 | 2515 | 76.94 (75.24-78.57) | 53.12 (51.48-54.75) | 0.04 (0.04-0.05) | 46.88 (45.25-48.52) | 0.77 (0.76-0.77) |
| Melanoma skin cancer | 1712 | 1223 | 97.06 (95.95-97.93) | 69.33 (67.09-71.51) | 0.00 (0.00-0.00) | 30.67 (28.49-32.91) | 0.85 (0.84-0.86) |
| Non-melanoma skin cancer | 1946 | 1082 | 49.72 (46.70-52.75) | 27.65 (25.67-29.69) | 0.04 (0.04-0.04) | 72.35 (70.31-74.33) | 0.64 (0.63-0.65) |
| Cancers of soft tissues | 229 | 239 | 72.80 (66.69-78.34) | 75.98 (69.91-81.36) | 0.00 (0.00-0.01) | 24.02 (18.64-30.09) | 0.88 (0.85-0.91) |
| Breast cancer | 7632 | 7755 | 95.46 (94.97-95.91) | 97.00 (96.59-97.37) | 0.03 (0.02-0.03) | 3.00 (2.63-3.41) | 0.98 (0.98-0.99) |
| Other female genital cancers | 2678 | 2808 | 91.42 (90.32-92.43) | 95.86 (95.03-96.58) | 0.02 (0.02-0.02) | 4.14 (3.42-4.97) | 0.98 (0.98-0.98) |
| Uterus | 1086 | 1088 | 96.32 (95.03-97.36) | 96.50 (95.23-97.51) | 0.00 (0.00-0.00) | 3.50 (2.49-4.77) | 0.98 (0.98-0.99) |
| Male genital cancers | 6544 | 5795 | 95.12 (94.53-95.66) | 84.23 (83.32-85.11) | 0.02 (0.02-0.02) | 15.77 (14.89-16.68) | 0.92 (0.92-0.93) |
| Prostate | 6198 | 5435 | 95.40 (94.81-95.94) | 83.66 (82.71-84.57) | 0.02 (0.02-0.02) | 16.34 (15.43-17.29) | 0.92 (0.91-0.92) |
| Cancers of urinary tract | 3883 | 3916 | 91.57 (90.66-92.42) | 92.35 (91.47-93.17) | 0.02 (0.02-0.03) | 7.65 (6.83-8.53) | 0.96 (0.96-0.97) |
| Kidney | 1012 | 1120 | 83.48 (81.18-85.61) | 92.39 (90.58-93.95) | 0.01 (0.01-0.02) | 7.61 (6.05-9.42) | 0.96 (0.95-0.97) |
| Bladder | 2786 | 2700 | 94.78 (93.87-95.59) | 91.85 (90.77-92.84) | 0.01 (0.01-0.01) | 8.15 (7.16-9.23) | 0.96 (0.95-0.96) |
| Cancers of central nervous system and eye | 1472 | 957 | 79.31 (76.60-81.84) | 51.56 (48.97-54.14) | 0.01 (0.01-0.02) | 48.44 (45.86-51.03) | 0.76 (0.74-0.77) |
| Cancers of endocrine glands | 1431 | 441 | 92.29 (89.39-94.60) | 28.44 (26.12-30.86) | 0.00 (0.00-0.00) | 71.56 (69.14-73.88) | 0.64 (0.63-0.65) |
| Hematologic malignancies | 2735 | 2780 | 87.63 (86.34-88.83) | 89.07 (87.84-90.21) | 0.02 (0.02-0.03) | 10.93 (9.79-12.16) | 0.95 (0.94-0.95) |
| Others | 997 | 1088 | 48.71 (45.70-51.73) | 53.16 (50.01-56.29) | 0.04 (0.04-0.04) | 46.84 (43.71-49.99) | 0.77 (0.75-0.78) |

^†^ If multiple records, we counted the first primary diagnosis of cancer for each patient.
